# Evolution of C, D and S-Type Cystatins in Mammals: An Extensive Gene Duplication in Primates

**DOI:** 10.1371/journal.pone.0109050

**Published:** 2014-10-17

**Authors:** Patrícia de Sousa-Pereira, Joana Abrantes, Ana Pinheiro, Bruno Colaço, Rui Vitorino, Pedro J. Esteves

**Affiliations:** 1 QOPNA, Centro de Espectrometria de Massa, Departamento de Química, Universidade de Aveiro, Aveiro, Portugal; 2 CIBIO, Centro de Investigação em Biodiversidade e Recursos Genéticos, InBio, Laboratório Associado, Universidade do Porto, Vairão, Portugal; 3 Departamento de Biologia, Faculdade de Ciências, Universidade do Porto, Porto, Portugal; 4 SaBio - IREC (CSIC-UCLM-JCCM), Ciudad Real, Spain; 5 Departamento de Ciências Veterinárias, CECAV, Universidade de Trás-os-Montes e Alto Douro, Vila Real, Portugal; 6 Centro de Investigação em Tecnologias da Saúde, IPSN, CESPU, Gandra, Portugal; University of Minnesota, United States of America

## Abstract

Cystatins are a family of inhibitors of cysteine peptidases that comprises the salivary cystatins (D and S-type cystatins) and cystatin C. These cystatins are encoded by a multigene family (*CST3*, *CST5*, *CST4*, *CST1* and *CST2*) organized in tandem in the human genome. Their presence and functional importance in human saliva has been reported, however the distribution of these proteins in other mammals is still unclear. Here, we performed a proteomic analysis of the saliva of several mammals and studied the evolution of this multigene family. The proteomic analysis detected S-type cystatins (S, SA, and SN) in human saliva and cystatin D in rat saliva. The evolutionary analysis showed that the cystatin C encoding gene is present in species of the most representative mammalian groups, i.e. Artiodactyla, Rodentia, Lagomorpha, Carnivora and Primates. On the other hand, D and S-type cystatins are mainly retrieved from Primates, and especially the evolution of S-type cystatins seems to be a dynamic process as seen in *Pongo abelii* genome where several copies of *CST1-like* gene (cystatin SN) were found. In Rodents, a group of cystatins previously identified as D and S has also evolved. Despite the high divergence of the amino acid sequence, their position in the phylogenetic tree and their genome organization suggests a common origin with those of the Primates. These results suggest that the D and S type cystatins have emerged before the mammalian radiation and were retained only in Primates and Rodents. Although the mechanisms driving the evolution of cystatins are unknown, it seems to be a dynamic process with several gene duplications evolving according to the birth-and-death model of evolution. The factors that led to the appearance of a group of saliva-specific cystatins in Primates and its rapid evolution remain undetermined, but may be associated with an adaptive advantage.

## Introduction

Saliva is an important body fluid that plays several roles in the oral cavity, participating in processes such as lubrication, digestion and protection of oral cavity [1]–[3]. In addition, saliva comprises the first line of defence of the oral cavity [1], [4]. Among the components responsible for this role there are several peptides, including cystatins. Cystatins belong to a superfamily of low molecular weight proteins that are involved in the inhibition of cysteine peptidases (CPs), such as mammalian cathepsins B, H and L [5]–[7]. Four main cystatin families are known: type-I cystatins or stefins; type-II cystatins; type-III cystatins or kininogens, and type-IV cystatins, or fetuins [8].

Cystatin C, D and S-type (S, SN and SA) are type-II cystatins composed by 120–125 amino acid residues containing two disulfide bonds. In humans, these cystatins are encoded by the genes *CST3*, *CST5*, *CST4*, *CST1* and *CST2*, respectively, which are located in tandem on chromosome 20 (20p11.21) [3], [8], [9]. Although cystatins C and D are frequently found in other body fluids, S-type cystatins are saliva-specific and share a high degree of amino acid similarity (∼88%) [8]. S-type cystatins act as cysteine-protease inhibitors, but they have also a small role in the regulation of salivary calcium and present antimicrobial activity [2], [8], [10], [11]. Not all type-II cystatins show the same activity, i.e. D and S-type cystatins are poorer inhibitors of cathepsins when compared to cystatin C while cystatin C is less effective in the bactericidal activity than cystatin S [8], [10], [12]. Additionally, human cystatin S presents high affinity to the tooth surface, which suggests a main role in the maintenance of the mineralized surfaces [8]. Indeed, there is a correlation between the activity of each cystatin and their evolution, with salivary cystatins becoming progressively less active against the host lysosomal cathepsins B, H, and L [8].

Multigene families arise by gene duplication and this is a useful mechanism to provide several copies of the same gene, and thus increase gene expression [13]. However, the copies might face pseudogenization, neofunctionalization and subfunctionalization [14]. Pseudogenization generally occurs when the copies present functional redundancy which frequently leads to the inactivation of one of the copies by accumulation of deleterious mutations and ultimately to the deletion from the genome [15]. In neofunctionalization both copies are maintained in the genome as new functions are acquired by one of the copies [16], [17]. In subfunctionalization there is a division of the ancestral functions between the two copies [18]. Furthermore, species-specific gene duplication might give rise to species-specific gene functions, contributing to species divergence and adaptation [13], [15].

For a long time it was generally accepted that evolution of multigene families was associated with processes of genetic exchange, such as gene conversion and unequal crossing-over that homogenize the sequences [19], [20]. This model of evolution was designated concerted evolution. The birth-and-death model of evolution [21]–[23] was later proposed to explain the pattern observed in the evolution of the immunoglobulins heavy chain variable region (*IGHV*), being quite similar to the accordion model proposed by Klein et al. [24] to explain the MHC evolution. The birth-and-death model of evolution suggests that during genome evolution, genes can either duplicate and be maintained very similar, diverge functionally or become pseudogenes. The end result of this process is a mixture of both divergent and highly homologous group of genes.

Previous evolutionary studies on this superfamily suggested that from the type-II cystatins here in study, cystatin C is the most ancestral, being found in several vertebrates. By duplication, cystatin C originated the ancestor of the subfamily of salivary cystatins (cystatin D, S, SA and SN) [8], [25], [26]. Several studies indicate that cystatin C had its origin in an ancestral cystatin at ∼650 million years ago (mya) during the evolution of bony vertebrates, while D and S-type cystatins have a more recent origin [8], [25]–[27]. Some authors argue that D and S-type cystatins evolved in Primates to protect the oral cavity from dietary and environmental CPs as they present poor inhibition for endogenous CPs [8]. However, some non-primate species have proteins also assigned as D or S-type cystatins, which does not make clear if the divergence of this specific group of cystatins occurred before or after the mammalian radiation. Furthermore, the evolution of these proteins in Primates is also not clear, making essential the understanding of their evolution to better interpret their function in the oral cavity. Here, we used a proteomic approach to assess the presence of these proteins in the saliva of several mammal species and conducted evolutionary analysis to determine their origin and shed light into their evolutionary history.

## Materials and Methods

### Samples

Dog (*Canis lupus*, mongrel breed), horse (*Equus cabalus*), cow (*Bos taurus*), sheep (*Ovis aries*) and human (*Homo sapiens*) saliva samples were collected during 4****minutes using salivettes (Sterile Saliva Collection Devices by Sarstedt) which were then centrifuged at 1000×g for 2****min to recover the saliva sample. For saliva collection in rat (*Rattus novergicus*, strain Wistar) and European rabbit (*Oryctolagus cuniculus*, domestic breed White New Zealand), the animals were first injected with the sialagogue pilocarpine (4 µmol/kg). All the samples were then centrifuged at 12000×g for 10****min at 4°C to collect the supernatant.

### Ethics statements

Human and animal saliva samples were collected using non-invasive methods and without provoking suffering. None of the animals included in this study was sacrificed and the fundamental ethical principles including human rights and animal welfare requirements at the national, regional and local regulations and directives were respected. Human volunteer donors gave their written informed consent after being informed of the research project’s nature and that the Declaration of Helsinki was respected. The ethics committee of Universidade de Trás-os-Montes e Alto Douro (UTAD), Portugal, that approved the human and animal studies was composed by Maria da Conceição Azevedo, José Luís Correia Ribeiro, Maria José Gomes and Pedro M. Mestre A. Silva. The animal studies were performed in the Departamento de Ciências Veterinárias, CECAV, Universidade de Trás-os-Montes e Alto Douro, Vila Real, Portugal.

### Proteomic analysis

Proteins present in saliva samples were separated by gel electrophoresis under denaturing conditions according to Laemmli [28]. For mass spectrometry analysis, protein bands located around the 15 kDa area were excised and were prepared according to the conditions reported elsewhere [29]. Mass spectra were obtained according to the conditions described by Vitorino et al. [29].

MS/MS data was searched against the Swissprot and NCBI protein databases for all species using paragon algorithm from ProteinPilot software (version 4.0, Applied Biosystems, USA) and Mascot software (v.2.1.0.4, Matrix Science Ltd, U.K.). An MS tolerance of 30 ppm was selected for precursor ions and 0.3 Da for fragment ions. Confidence levels ≥99% were used as positive protein identification criteria. In order to estimate the false discovery rate (FDR) a reverse decoy database was created for all SwissProt resulting in 5% of FDR [false positive peptides/(false positive peptides + total peptides)]*100. Unique peptides retrieved from FDR search were considered for analysis.

### Evolutionary analysis

Mammalian cystatins C, D, S, SA and SN nucleotide coding sequences were obtained from NCBI (http://www.ncbi.nlm.nih.gov) and Ensembl (http://www.ensembl.org) databases. Only coding and complete sequences were considered. Accession numbers for all retrieved sequences are presented in table S1.

Multiple sequence alignments were performed in BioEdit Sequence Alignment Editor [30] using ClustalW [31] followed by manual correction. *Gallus gallus CST3* and *Columba livia CST-like* sequences were included as outgroup. To assess the fit of our dataset to 88 models of nucleotide evolution, the jModelTest v2.1.1 [32] was used considering the Akaike information criterion (AIC). Evolutionary relationships between the type-II cystatins were analysed in Maximum Likelihood (ML) and Bayesian inference (BI) frameworks. ML analyses were performed using PhyML v3.0 [33] specifying TPM3+I+G as the best fitting mutation model. The support of the resulting nodes was estimated using 1000 bootstrap replicates as implemented in PhyML. BI analyses were performed using MrBayes version 3.1.2 [34], [35], using a general time reversible (GTR) model of nucleotide substitutions with invariant gamma distribution (4 categories). Markov Chain Monte Carlo (MCMC) chains run for 2×10^6^ generations, sampling every 1000 generations, with the first 200000 sampled trees discarded. Convergence was checked using Tracer v1.5 [36] and the tree was visualized with FigTree v1.4.0 (http://tree.bio.ed.ac.uk/software/figtree/).

To evaluate the selective pressures on the *CST3* gene, five methods available in the DATAMONKEY web server [37] were used: SLAC, FEL, REL, MEME and FUBAR. For these analyses, the best fitting nucleotide substitution model was determined through the automatic model selection tool available on the server. For SLAC, FEL and MEME the P-values were set into 0.05; for REL, a P-value of 100 was used and for FUBAR a P-value of 0.95.

## Results

### Proteomic analysis

Characterization of the saliva from different mammal species by a proteomic approach did not allow the identification of cystatin C in any of the species studied. S-type cystatins (S, SA and SN) were only found in human saliva and cystatin D was identified only in rat saliva (Table 1).

**Table 1:** Cystatins found by LC-MS/MS from human, dog, sheep, cattle, horse, rabbit and rat saliva.

	Cystatin SN	Cystatin SA	Cystatin S	Cystatin D	Cystatin C
Human	CYTN_HUMAN	gi|235948	CYTS_HUMAN	-	-
Dog	-	-	-	-	-
Bovine	-	-	-	-	-
Sheep	-	-	-	-	-
Horse	-	-	-	-	-
Rabbit	-	-	-	-	-
Rat	-	-	-	gi|344250777	-

The searches that allowed these identifications were made using the Mascot software against the Swissprot and NCBI databases. The accession numbers/gi numbers are indicated.

### Evolutionary analysis

The sequences of the genes encoding C, D and S-type cystatins (*CST3*, *CST5*, *CST4*, *CST1* and *CST2*) retrieved from the public databases showed that cystatin C had already been annotated in several mammal species while the sequences assigned as D and S-type cystatins were almost exclusive to Primates. However, in some cases, the available sequences were incorrectly annotated or barely assembled.

**Figure 1:**
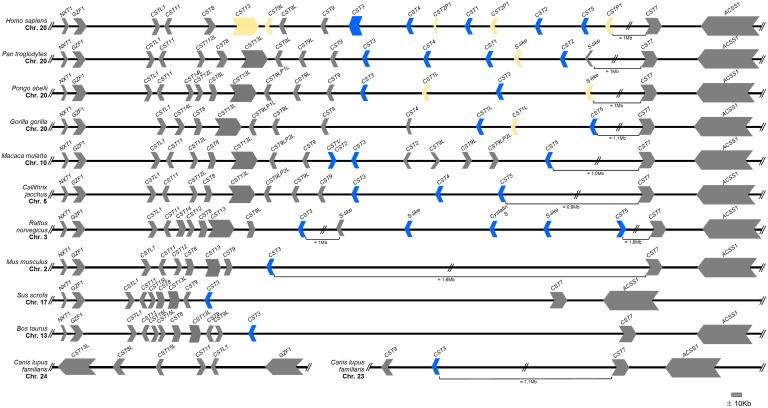
Type-II cystatin gene cluster region overview. Organization of the *CST1*-*5* genes in human (*Homo sapiens*), chimpanzee (*Pan troglodytes*), orangutan (*Pongo abelii*), gorilla (*Gorilla gorilla*), rhesus monkey (*Macaca mulatta*), marmoset (*Callithrix jacchus*), rat (*Rattus norvegicus*), mouse (*Mus musculus*), pig (*Sus scrofa*), cattle (*Bos taurus*) and dog (*Canis lupus familiaris*). The transcriptional orientation of the genes is shown; the pseudogenes are highlighted in yellow (data from ENSEMBL and NCBI databases). The genes used in the subsequent analysis are highlighted in blue.

In humans, the genomic location of the genes encoding these proteins is well characterized. Indeed, they are located in tandem on chromosome 20 in the locus p11.21. In other mammal species, we observed that the locus containing these genes is often located in a syntenic region, being flanked by the *NXT1* and *GZF1* genes at the 5′ end and the *ACSS1* gene at the 3′ end (Figure 1). In dog genome, the syntenic region is not conserved since this region seems to have been split into chromosomes 23 and 24. However, cystatin genes appear in the same order as observed in the other mammals’ genomes. Along with the *CST1-5* genes, other type-II cystatin genes locate to this syntenic region, including *CSTL1*, *CST11*, *CST8*, *CST9L*, *CST9* and *CST7* (Figure 1).

*CST3* is the only gene present in all the mammalian orders included in this study while the remaining analysed genes, *CST1*, *CST2*, *CST4* and *CST5*, are present mostly in Primate genomes. On the human genome the five studied genes are arranged as follows: *CST3-CST4-CST1-CST2-CST5*, spanning ∼200 kb at approximate equal distance (Figure 1). A similar organization was also observed in the *Pan troglodytes* genome, despite the lack of *CST5*, and in the *Gorilla gorilla* genome, despite the lack of *CST3* and *CST2* genes. Multiple copies of a *CST1-like* gene have been annotated in the *Pongo abelii* genome; all, but *CST1-like* (3) (XM_002834995) for which the chromosomal location remains unknown, locate on chromosome 20 at random positions.

In the *Macaca mulatta* cystatin locus, located on chromosome 10 (and not chromosome 20 as for Humans and Apes), the *CST1/CST2* gene (annotated as *CST1* in ENSEMBL and as *CST2* in NCBI) is located in an unusual position within *CST9* and *CST3* genes; in addition, downstream of the *CST5* gene, where S-type cystatins genes were expected to be located, more than one copy of the *CST9L* gene is found. Moreover, the *CST1/CST2* gene and the *CST9L* genes present a different genome transcriptional orientation from that of their orthologues.

In *Rattus norvegicus*, downstream to the *CST3* gene, four coding genes are annotated as S or S-like cystatins followed by one gene assigned as *CST5*. This chromosomal organization is similar, but not entirely consistent with that observed in Primates. In the remaining mammals, representing the Artiodactyla (*Bos taurus* and *Sus scrofa*) and Carnivora (*Canis lupus*) orders, only the *CST3* gene could be identified.

**Figure 2:**
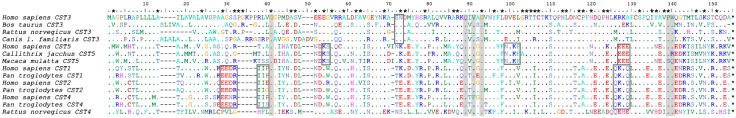
Amino acid composition of cystatins. *Homo sapiens* cystatin C (P01034); *Bos taurus* cystatin C (P01035); *Rattus norvegicus* cystatin C (P14841); *Canis lupus familiaris* cystatin C (J9NS29); *Homo sapiens* cystatin D (P28325); *Callithrix jacchus* cystatin D (ENSCJAP00000001156); *Macaca mulatta* cystatin D (G7N352); *Homo sapiens* cystatin SN (P01037); *Pan troglodytes* cystatin SN (H2QK35); *Homo sapiens* cystatin S (P01036); *Pan troglodytes* cystatin S (H2QK34); *Homo sapiens* cystatin SA (P09228); *Pan troglodytes* cystatin SA (H2QK36) and *Rattus norvegicus* cystatin S (P19313). Filled grey boxes indicate conserved amino acid motifs; empty boxes indicate conserved amino acids characteristic of each cystatin; asterisks (*) mark the codons on *CST3* under negative selection.

The alignment of the amino acid sequences of C, D and S-type cystatins allowed the detection of amino acid motifs that could be relevant to their functional role (Figure 2). For cystatins, three motifs that are important for the inhibition of cysteine peptidases (CPs) have been described: one N-terminal G residue and the QXVXG and PW motifs [8]; these were observed in almost every cystatin sequence retrieved (Figure 2). Moreover, by looking at the amino acid sequences, the different cystatins present specific amino acid motifs that allow their distinction (Figure 2). However, the S-type cystatins, S, SA and SN, share several amino acid substitutions that hamper their assignment as different types. From the amino acid alignment it was also noticeable that the rat cystatin S is highly divergent, being substantially different from their Primates’ counterparts. The tests for selective pressures for cystatin C showed that only ∼30% of randomly positioned codons are under negative selection (Figure 2).

**Figure 3:**
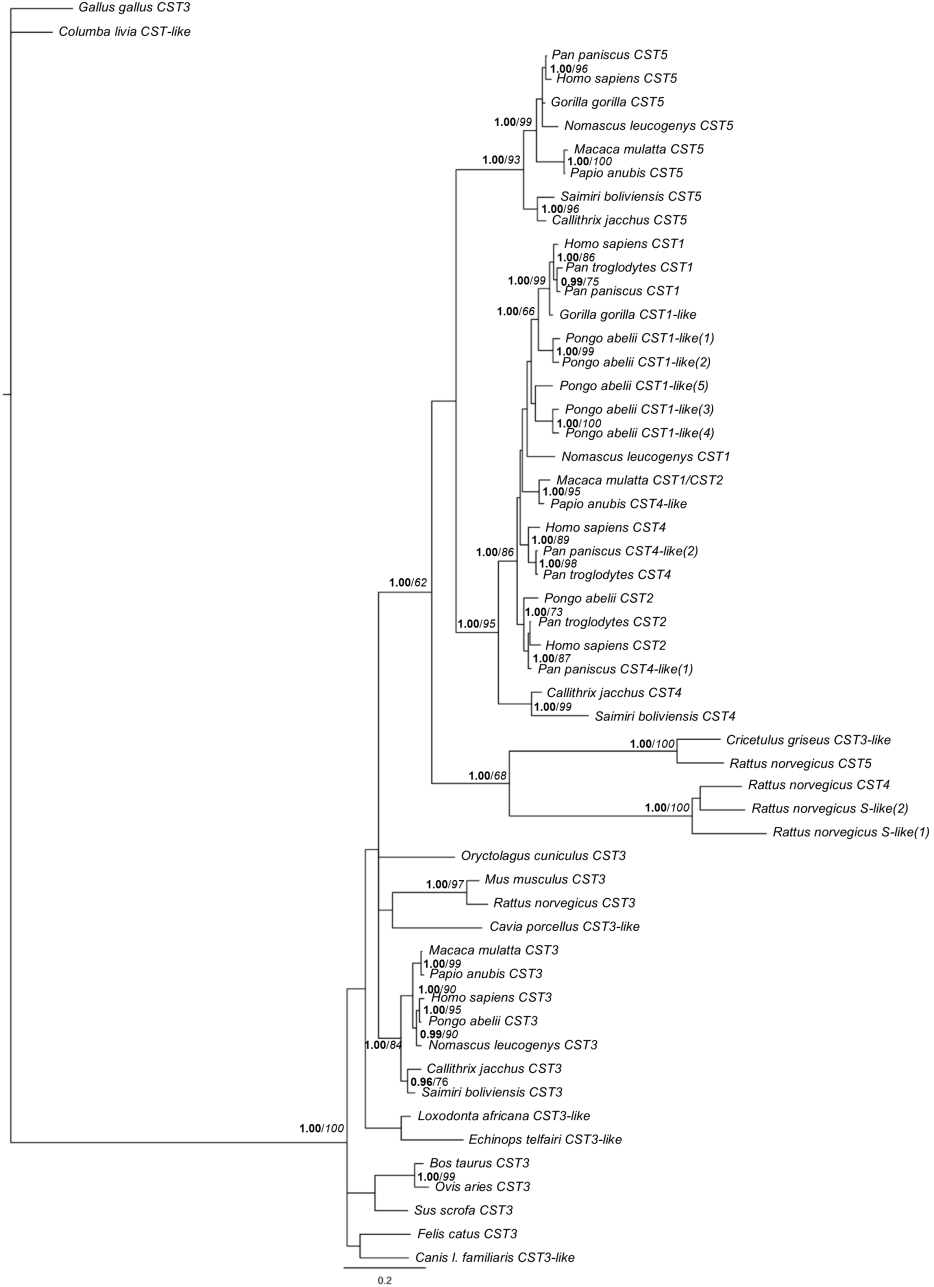
Phylogenetic tree inferred by using Maximum Likelihood (ML) and Bayesian inference (BI). TPM3+I+G was the best fitting mutation model. For ML 1000 bootstrap replicates were considered and for BI posterior probabilities were calculated; posterior probabilities (**bold**) over 0.95 and bootstrap confidence (*italic*) over 90% are considered valid support and are shown in the tree.

The ML and BI methodologies used to study the evolutionary relationships between these type-II cystatins returned phylogenies with similar topologies (Figure 3). Cystatin C encoding sequences (*CST3* gene) of representatives of several mammal orders are at a basal position in the tree, within which the branching generally agrees with the accepted mammalian phylogeny [38]. All the Primates’ D and S-type cystatins and the Rodents’ cystatins are grouped in a branch well supported by the Bayesian analysis (1.00 posterior probability). Primates’ cystatin D and S-type cystatins (*CST1*, *CST2* and *CST4*) form a poorly supported group (posterior probability and bootstrap confidence under 0.95 and 90%, respectively). Within the Primates’ cystatin D group, sequences cluster in accordance to the Primates’ phylogeny [39]. As for S-type cystatins, this clustering is not clear, with the relationships among S, SN and SA cystatins not well resolved. Within the S-type group, the sequences of an S-like cystatin from New World Monkeys (Platyrrhini) form a highly-supported group (1.00 posterior probability, 99% bootstrap confidence). For Catarrhini (Apes and Old World Monkeys), the branches containing SA cystatins and S cystatins are well supported (1.00 posterior probability for both) and seem to have resulted from the duplication of an ancestral gene. Albeit poorly supported, the five copies of a *CST1-like* that are annotated in the *Pongo abelii* genome (*CST1-like*(1) to *CST1-like*(5)), group in the cystatin SN group indicating that these are *CST1* genes. These copies do not group together suggesting at least two major duplication events in their origin. The Rodent sequences previously identified as D and S cystatins and *Cricetulus griseus* C-like cystatin form a group apart from all other D and S-type cystatins (1.00 posterior probability; 68% bootstrap confidence), reflecting the high genetic distances between these sequences and that of other Primates’ cystatins.

With this phylogenetic analysis some discrepancies in the annotations of the different databases were detected. For example, *Macaca mulatta* ENSEMBL *CST1* sequence (ENSMMUT00000005570) matches the NCBI database *CST2* sequence (XM_001097284). These conflicts in gene annotation might result from the high similarity between S-type cystatins.

## Discussion

Despite the importance of salivary cystatins in the protection of the human oral cavity, they are regularly described as Primate specific. The proteomic approach used in the analysis of rat, dog, rabbit, sheep, horse, bovine and human saliva only revealed the presence of cystatin D in rat saliva and S-type cystatins in human saliva. None of the other type-II cystatins were found in these species. However, presence of cystatin C in human saliva had been previously confirmed [40], despite not being a major cystatin in this fluid [41]. Since high salivary levels of cystatin C are mainly associated with oral inflammatory processes [42], the presence of low amounts of cystatin C can explain its non-detection by the proteomic approach.

Sequences of the *CST3* gene are present in almost all of the analysed genomes, consistently located upstream of the *CSTL1*, *CST11*, *CST9L*, *CST9* and *CST8* genes which are other type-II cystatins genes also located in this cystatin locus (Figure 1). Besides its location in a syntenic region, the retrieved cystatin C (*CST3*) amino acid sequences showed that cystatin domains _55_QXVXG_59_, _105_PW_106_ and the N-terminal G_11_ [25] are highly conserved in almost all sequences analysed (Figure 2). Thus, the absence of a high degree of differentiation between cystatin C proteins in the different mammal species may suggest an important role of this protein in mammals that has been maintained in mammalian evolution. In addition, all cystatin C sequences clustered in a well-supported group in the phylogenetic tree and further grouped in accordance to the accepted molecular tree of placental mammals, clearly distinguishing branches for Primates, Lagomorphs, Rodents, Carnivores and Artiodactyls (Figure 3), thus reflecting the mammalian evolution [38]. This, and the basal position of these sequences in the tree, support the previous hypothesis that *CST3* is the most ancestral gene among the genes here in study [8], [26].

On the constructed ML and BI phylogenetic trees, the Primates’ cystatins D (*CST5*) appear on a single highly-supported cluster, comprising sequences from Platyrrhini (New World Monkeys) and Catarrhini (Old World Monkeys, Great Apes and Human), suggesting that cystatin D originated in the ancestor of the Simiiformes at ∼36–50 mya [39]. Moreover, the phylogenetic relationships between the *CST5* sequences follow these species accepted phylogeny [39].

The remaining genes, *CST1*, *CST2* and *CST4*, which encode type-S cystatins (SN, SA and S, respectively) were found in several Primates’ genomes, but the high degree of similarity between them might confound their identification. In the phylogenetic tree, these sequences appear in a highly-supported branch (1.00 posterior probability, 95% bootstrap confidence), which supports a common origin for these Simiiformes’ S-type cystatins [39] (Figure 3), but the branching within the S-type cystatins is not as well resolved. The Platyrrhini (New World monkeys) cystatins are clustered in a well-supported branch in a basal position. As for SA cystatins, its presence is only confirmed in the Hominidae family. In this branch, *Pan paniscus CST4-like(1)* groups with *Homo sapiens CST2*, *Pan troglodytes CST2* and *Pongo abelli CST2*, which suggests an incorrect annotation of this sequence; due to its similarity to other *CST2* genes, this sequence is most likely of a *CST2* gene. The tree also presents a branch containing Old World monkeys’ cystatins (*Macaca mulatta CST1/CST2* and *Papio anubis CST4*-like). The information from the chromosomal location of the gene assigned as *CST1/CST2* in *Macaca mulatta* places it in an unusual region compared to that of the other S-type cystatins (Figure 1); however the phenomenon that led to the misconfiguration of this chromosomal region is unclear. Its location in the tree, together with the *Papio anubis CST4-like*, suggests that this branch represents the S-type cystatins in Old World monkeys which has not suffered further duplications (Figure 3).

**Figure 4:**
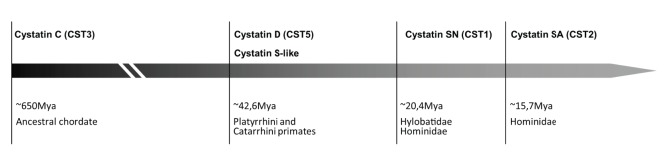
Diagram of salivary cystatin evolution. Cystatin genes estimated emergence is shown (divergence times were based on [45]).

According to our results, an ancestral S-like gene appeared in the Simiiformes common ancestor and persisted in Platyrrhini where no further duplication occurred. In Catarrhini, this ancestral S-like gene gave rise to S-type Old World monkeys cystatins and in Apes the ancestral gene evolved and duplicated giving rise to *CST1*, *CST2* and *CST4* (Figure 4).

The evolution of S-type cystatins through duplication events in Apes seems to be an ongoing process as observed from the *Pongo abelii* genome where several sequences of cystatin SN (*CST1*-*like* gene) were found. These *Pongo abelii CST1-like*(1–5) copies are separated into two groups, one group is represented by *CST1-like*(1) and (2), and the other contains *CST1-like*(3), (4) and (5); this indicates an origin through at least two independent duplications. However, further studies are necessary to determine whether all *CST1-like* copies from *Pongo abelii* are equally transcribed. In these duplicates, several amino acid substitutions occur in the two characteristic cystatin conserved domains (data not shown), but it is not clear if the observed mutations can interfere with the cysteine protease inhibitor activity or if they involve the acquisition of novel functions.

In Rodents, the phylogenetic position of the *CST3*-*like* gene in *Cricetulus griseus* and the *CST5* and *CST4-like* genes in *Rattus norvegicus*, along with their genomic location which resembles the gene organization observed in Primates (Figure 1), could imply a common origin for these cystatins. Furthermore, the proteomic analysis revealed that rat cystatin D is expressed in saliva, as for S-type cystatins in Primates (Table 1). The rat cystatin S was formerly identified as LM protein, being latter assigned to cystatin S due to similarities of the amino acid sequence with other type-II cystatins (Figure 2) and to its ability to inhibit some cysteine proteases [43]. The Rodent cystatins retrieved present similar functional domains to that of the type-II cystatins (http://prosite.expasy.org/); nevertheless, while for human cystatin S its functions are defined [8], these have not been determined for the Rodent proteins. Thus, our results suggest that D and S like cystatin genes emerged before the mammalian radiation but only persisted in Primates and in Rodents.

Multigene families are the result of gene duplication and according to the birth-and-death evolution model some of the copies might remain similar, some might diverge functionally and others might become pseudogenes [18]. Evolution of cystatins seems to follow this model with many proteins presenting similar structure and function. A few pseudogenes have been identified [44] (Figure 1), but some novel functions such as antiviral and antibacterial activities have also been attributed to some members, which might explain their persistence [8]. The factors that lead to the appearance of a group of saliva-specific cystatins in Primates and its rapid evolution remain undetermined, but might be associated with an adaptive advantage.

## Conclusions

Salivary cystatins (D and S-type cystatins) play a crucial role in human saliva for homeostasis of the oral cavity. The main function of this protein family is the inhibition of CPs, but new roles, such as antimicrobial activity, have been detected. This neofunctionalization might explain the maintenance of a high number of copies for some of these genes in some species. In human saliva these cystatins can be found, but screening the saliva from other mammalian species showed that cystatin D was found only in rat saliva while none of the other salivary cystatins were present. Although the mechanisms that lead to the evolution of cystatins are unknown, Primates’ cystatins compose a distinct group that arose by several rounds of duplication with the proteins most likely acquiring new functions. In particular for S-type cystatins, their high similarity and the lack of specific characteristics that allow their distinction frequently lead to their misannotation in the databases. In Rodents, a group of cystatins has also evolved with similar genomic location and conserved domains when comparing to the Primates’ D and S-type cystatins; its location in the phylogenetic tree suggests a common origin. These results show that these genes were present in the genome before the mammalian radiation.

## Supporting Information

Table S1 **Accession numbers of the cystatin nucleotide sequences used in the phylogenetic analysis.**
(DOCX)

Alignment S1 **Alignment used to conduct all the analysis.**
(FAS)
