# Supplementary material for: Evolution of C, D and S-Type Cystatins in Mammals: An Extensive Gene Duplication in Primates
Source: PLoS One. 2014 Oct 17;9(10):e109050. doi: 10.1371/journal.pone.0109050 (PMC4201479; doi:10.1371/journal.pone.0109050)
Supplement: Table S1 — Accession numbers of the cystatin nucleotide sequences used in the phylogenetic analysis. (DOCX) [file pone.0109050.s001.docx]

|  | **Species** | **Accession number** | **Chromosome** |
| --- | --- | --- | --- |
| **Cystatin C** | *Bos taurus CST3* | NM_174029 | 13 |
|  | *Callithrix jacchus CST3* | XM_002747514 | 5 |
|  | *Canis lupus familiaris CST3-like* | XM_003639821 | 23 |
|  | *Cavia porcellus CST3-like* | XM_003476403 | unknown |
|  | *Cricetulus griseus CST3-like* | XM_003499974 | unknown |
|  | *Echinops telfairi CST3-like* | XM_004715300 | unknown |
|  | *Felis catus CST3* | NM_001184972 | A3 |
|  | *Homo sapiens CST3* | NM_000099 | 20 |
|  | *Loxodonta africana CST3-like* | XM_003411620 | unknown |
|  | *Macaca mulatta CST3* | NM_001032924 | 10 |
|  | *Mus musculus CST3* | NM_009976 | 2 |
|  | *Nomascus leucogenys CST3* | XM_003280847 | 13 |
|  | *Oryctolagus cuniculus CST3* | NM_001082706 | 4 |
|  | *Ovis aries CST3* | JX534543 | 13 |
|  | *Papio anubis CST3* | XM_003905160 | 10 |
|  | *Pongo abelii CST3* | ENSPPYT00000012515 | 20 |
|  | *Rattus norvegicus CST3* | NM_012837 | 3 |
|  | *Saimiri boliviensis CST3* | XM_003942920 | unknown |
|  | *Sus scrofa CST3* | NM_001044602 | 17 |
| **Cystatin D** | *Callithrix jachus CST5* | ENSCJAT00000001226 | 5 |
|  | *Gorilla gorila CST5* | XM_004061910 | 20 |
|  | *Homo sapiens CST5* | NM_001900 | 20 |
|  | *Macaca mulatta CST5* | XM_001097898 | 10 |
|  | *Nomascus leucogenys CST5* | XM_003278498 | 13 |
|  | *Pan paniscus CST5* | XM_003829361 | unknown |
|  | *Papio anubis CST5* | XM_003905168 | 10 |
|  | *Rattus norvegicus CST5* | NM_001108961 | 3 |
|  | *Saimiri boliviensis CST5* | XM_003942944 | unknown |
| **Type-S Cystatins** | *Gorilla gorila CST1-like* | XM_004061909 | 20 |
|  | *Homo sapiens CST1* | NM_001898 | 20 |
|  | *Nomascus leucogenys CST1* | XM_003278497 | 13 |
|  | *Pan paniscus CST1* | XM_003829363 | unknown |
|  | *Pan troglodytes CST1* | XM_001147668 | 20 |
|  | *Pongo abelii (1) CST1-like* | XM_002834542 | 20 |
|  | *Pongo abelii (2) CST1-like* | XM_002834568 | 20 |
|  | *Pongo abelii (3) CST1-like* | XM_002834995 | unknown |
|  | *Pongo abelii (4) CST1-like* | XM_002834557 | 20 |
|  | *Pongo abelii (5) CST1-like* | XM_002834559 | 20 |
|  | *Macaca mulatta CST1/CST2* | ENSMMUT00000005570 / XM_001097284 | 10 |
|  | *Pongo abelii CST2* | XM_002830032 | 20 |
|  | *Homo sapiens CST2* | NM_001322 | 20 |
|  | *Pan troglodytes CST2* | XM_001147822 | 20 |
|  | *Callithrix jachus CST4* | XM_002747520 | 5 |
|  | *Homo sapiens CST4* | NM_001899 | 20 |
|  | *Pan paniscus CST4-like(1)* | XM_003829362 | unknown |
|  | *Pan paniscus CST4-like(2)* | XM_003829364 | unknown |
|  | *Pan troglodytes CST4* | XM_514553 | 20 |
|  | *Papio anubis CST4-like* | XM_003905164 | 10 |
|  | *Saimiri boliviensis CST4* | XM_003945122 | unknown |
|  | *Rattus norvegicus CST4* | [NM_198685](http://www.ncbi.nlm.nih.gov/nuccore/NM_198685.1) | 3 |
|  | *Rattus norvegicus S-like(1)* | [NM_001037350](http://www.ncbi.nlm.nih.gov/nuccore/NM_001037350.1) | 3 |
|  | *Rattus norvegicus S-like(2)* | [XM_003749587](http://www.ncbi.nlm.nih.gov/nuccore/XM_003749587.1) | 3 |
| **Outgroup** | *Gallus gallus CST3* | NM_205500 | 3 |
|  | *Columba livia CST-like* | XM_005508801 | unknown |
